# Alzheimer's disease: the amyloid hypothesis and the Inverse Warburg effect

**DOI:** 10.3389/fphys.2014.00522

**Published:** 2015-01-14

**Authors:** Lloyd A. Demetrius, Pierre J. Magistretti, Luc Pellerin

**Affiliations:** ^1^Department of Organismic and Evolutionary Biology, Harvard UniversityCambridge, MA, USA; ^2^Max Planck Institute for Molecular GeneticsBerlin, Germany; ^3^Division of Biological and Environmental Sciences and Engineering, King Abdullah University of Science and TechnologyThuwal, Saudi Arabia; ^4^Laboratory of Neuroenergetics and Cellular Dynamics, Brain Mind Institute, Ecole Polytechnique Fédérale de LausanneLausanne, Switzerland; ^5^Laboratory of Neuroenergetics, Department of Physiology, University of LausanneLausanne, Switzerland

**Keywords:** age-related disease, mitochondrial dysregulation, metabolic alteration, the Inverse Warburg effect, inverse cancer comorbidity

## Abstract

Epidemiological and biochemical studies show that the sporadic forms of Alzheimer's disease (AD) are characterized by the following hallmarks: (a) An exponential increase with age; (b) Selective neuronal vulnerability; (c) Inverse cancer comorbidity. The present article appeals to these hallmarks to evaluate and contrast two competing models of AD: the amyloid hypothesis (a neuron-centric mechanism) and the Inverse Warburg hypothesis (a neuron-astrocytic mechanism). We show that these three hallmarks of AD conflict with the amyloid hypothesis, but are consistent with the Inverse Warburg hypothesis, a bioenergetic model which postulates that AD is the result of a cascade of three events—mitochondrial dysregulation, metabolic reprogramming (the Inverse Warburg effect), and natural selection. We also provide an explanation for the failures of the clinical trials based on amyloid immunization, and we propose a new class of therapeutic strategies consistent with the neuroenergetic selection model.

## Introduction

Epidemiological studies of the incidence of Alzheimer's disease (AD) distinguish between two classes of individuals with clinical and histopathological features of the disorder—those with an autosomal dominant inheritance—an early onset group; and those with the sporadic form of the disease—a late onset group.

Investigators have observed genetic defects in individuals with the familial forms of the disease. The genes implicated are the amyloid precursor protein (APP), and the secretases, presenilin 1 and presenilin 2, enzymes involved with APP processing. Mutant forms of these genes induce an overproduction of beta amyloid due to an alteration in APP processing, creating an imbalance between production and clearance, and the clinical and histopathological phenotype associated with AD. This correlation between beta amyloid and the various histological and clinical hallmarks of the disease is the basis for the Amyloid hypothesis as a model for the familial forms of AD. The model, first proposed by Glenner and Wong (1984), contends that the neurodegenerative disease is due to an imbalance between the generation and clearance of beta amyloid.

The hypothesis of Glenner and Wong was subsequently shown to be consistent with certain molecular, biochemical and neuropathological studies of sporadic forms of AD (Selkoe, 1991; Hardy and Selkoe, 2002; Hardy, 2009). In parallel, the demonstration that the Aβ peptide exhibits neurotoxicity has led to the development of an important area of research around this topic as the main explanation for the etiology of the disease (Cavallucci et al., 2012). Accordingly, the amyloid model emerged as the organizing element in studies of both familial and sporadic forms of AD.

The reviews by Bertram and Tanzi (2008) as well as Tanzi and Bertram (2005) have elucidated the genetic origins for the familial and the late onset forms of the disease. These articles have documented and analyzed genes that are considered potential risk factors for AD. These studies, however, indicate that none of the genes implicated in the familial form of AD consistently influences disease risk in the late onset form. In the case of one genetic variant, an allele of the apolipoprotein E gene (APOE) called APOE4, it was shown that it represents a risk factor, but it accounts for very little in the heritability of the disease and cannot be considered as a cause of the early onset form of the disease.

Indeed, biochemical and epidemiological studies now indicate that age is the dominant risk factor in sporadic forms of AD. These investigations suggest that the amyloid plaques that are considered the biochemical hallmarks of the disease are also consistent with an age-related misfolding of the APP protein (Chiti and Dobson, 2009). However, in spite of these studies, proponents of the amyloid hypothesis have maintained that a mutation induced overproduction of beta amyloid underlies both the early onset and the late onset forms of AD, and consequently, both forms of the disease can be ascribed similar etiologies (Hardy, 2009; Selkoe, 2012).

The extrapolation of the model of early onset forms of AD to the late onset expressions is based on the assumption that age is primarily a measure of how long it takes for neurons to accumulate toxic moieties of beta amyloid. Accordingly, an imbalance between amyloid production and clearance can also be considered as the primary cause of sporadic AD. This argument is the rationale for therapeutic strategies of late onset AD based on targeting amyloid pathways, either through passive immunotherapy against beta amyloid, or active inhibition of beta amyloid generation (Hardy and Selkoe, 2002; Hardy, 2009).

The amyloid cascade model enjoys a dominant position in studies of neurodegeneration. There is, however, some dissent. This stems primarily from numerous conflicts between empirical observations and the predictions of the model. Some of the most prominent anomalies are as follows (see also Drachman, 2006; Giannakopoulos et al., 2009; Pimpliker, 2009; Nelson et al., 2012):

- Neuritic plaques continuously occur in the brain of cognitively unimpaired individuals.
- There is a weak correlation between the density of plaques and the degree of dementia.
- Cognitively intact individuals have a significantly large incidence of pre or post-mortem detected amyloid plaques.

These anomalies, and the consistent failure of clinical trials designed to assess the efficacy of therapeutic strategies, have stimulated various efforts to modify the premises of the model. However, the amendments proposed (see Hardy, 2009) for an evaluation, are all within the neuron-centric framework of amyloid production and clearance. Hence, they do not represent a significant advance in our understanding of the molecular basis of the sporadic forms of AD.

The amyloid hypothesis considers the sporadic forms of AD, as a disease determined primarily by the instability of the nuclear genome. The gene centered model essentially ignores the effect of two factors, energy and age, which play critical roles in neurodegenerative diseases.

Energy is the primary determinant of neuronal viability. Defects in energy metabolism may lead to a failure in the maintenance and restoration of ion gradients associated with synaptic transmission. In neurons, mitochondria are the main energy producing organelles. The mitochondria generate energy by oxidizing carbons derived from dietary carbohydrates and fat to generate heat and ATP (Nicholls and Ferguson, 2002; Wallace et al., 2010). The coherence of this process is determined by the coupling efficiency of oxidative phosphorylation. Age is the critical determinant of the efficiency of energy production. Aging at the molecular level is associated with the increase in molecular disorder induced by random perturbations in the activity of large biomolecules (see below for a description). These changes will ultimately result in a decline in the coupling efficiency of oxidative phosphorylation, and metabolic dysregulation (Hayflick, 2007a; Demetrius, 2013).

Energy metabolism is altered by age. Cellular metabolism invokes not only oxidative phosphorylation, an electrochemical process, but also glycolysis, a chemical process. There is no single gene for either of these processes, although genes do encode the individual enzymes involved in both metabolic pathways. Energy transduction by means of glycolysis is determined by a sequence of chemical reactions which are localized in the cytosol. In the case of oxidative phosphorylation, energy transduction is contingent on two processes, the pumping of protons out of the mitochondrial inner membrane by the electron transport chain, and the conversion of proton flow into ATP (Lehninger, 1965).

The efficiency of these two processes is highly dependent on the activity of the enzymes involved in the metabolic reactions. Enzyme activity will decline with age in view of the intrinsic thermodynamic instability of large biomolecules. This instability will result in a loss of molecular fidelity and an impairment in the capacity of the cells to appropriate energy from the external environment and to convert this energy into biosynthetic work. Consequently, the capacity of neurons to convert substrates such as glucose and lactate into ATP and to use this energy to maintain neuronal viability will also decline with age.

The model for the sporadic forms of AD proposed in Demetrius and Simon (2012) as well as in Demetrius and Driver (2013), implicates these two factors, energy and age, as the critical elements in the origin of neurodegenerative diseases.

This neuroenergetic perspective posits that the primary cause of sporadic forms of AD is an age-induced energy deficit in the mitochondrial activity of neurons, and the up-regulation of oxidative phosphorylation as a compensatory mechanism of energy production to maintain the viability of the impaired cells.

The model contends that an age-induced mitochondrial dysfunction will initiate the following cascade of events which ultimately results in neuronal loss and dementia:

- *Metabolic alteration:* A compensatory increase in oxidative phosphorylation in order to maintain adequate energy production, and thereby ensure neuronal viability.
- *Natural selection:* Competition for oxidative energy substrates between healthy neurons, utilizing the standard modes of energy production, and mildly impaired neurons, defined by compensatory increases in OxPhos activity.
- *Disease propagation:* The spread of metabolic abnormalities within the brain due to the selective advantage which increased OxPhos activity confers to cells in the cerebral microenvironment.

The model for the origin of cancer proposed by Warburg et al. (1924) postulates that cancer is a metabolic disease initiated by mitochondrial abnormalities, and subsequent metabolic alteration to compensate for the diminished energy induced by impaired mitochondria. The metabolic alteration proposed in this model is the up-regulation of glycolytic activity. This mode of metabolic reprogramming, now known as the Warburg effect, is a well-established phenomenon in studies of the etiology and proliferation of cancer. This is indicated by the widespread clinical use of “^18^Fluorodeoxyglucose” position emission tomography as a diagnostic marker for certain types of cancer.

Oxidative phosphorylation and glycolysis are complementary mechanisms which cells utilize to meet their energy demands. We have therefore called the up-regulation of OxPhos activity, which we claim underlies the origin of sporadic forms of AD, the Inverse Warburg effect.

The cornerstone of the neuroenergetic perspective is the competition for oxidative energy substrates between healthy neurons, that is neurons with normal OxPhos activity, and impaired neurons, cells with up-regulated OxPhos activity. The outcome of this competition depends on the relative capacity of the two types of neurons to appropriate energy substrates, and to convert these substrates into ATP. This capacity is quantitatively described by the statistical measure, evolutionary entropy, a measure of the number of pathways of energy flow within a metabolic network (Demetrius, 1997, 2013).

This paper will review the theoretical and empirical support for the Inverse Warburg hypothesis. We will re-evaluate the amyloid hypothesis by contrasting its tenets with the principles underlying the Inverse Warburg hypothesis. The contrast between the two classes of models rests on the following three criteria which characterize certain cellular, demographic and epidemiological features of sporadic forms of AD.

- *Selective neuronal vulnerability*—Neurons differ in terms of their vulnerability to AD: The neurons providing the projection from entorhinal cortex to the dentate gyrus and the pyramidal cells are the most vulnerable cell types. The difference in vulnerability reflects the usual course of the disease in which episodic memory is the function affected in the early stages of AD (Hof and Morrison, 2004).
- *Age as a risk factor*—The Darwinian fitness of an organism, that is the capacity of the organism to contribute to the ancestry of future generations is highly dependent on age. Up to the age of reproductive maturity, there will be intense selection to maintain the viability of the organism. Such a maintenance increases the long term contribution of the organism to the ancestry of successive generations. After the age of reproductive maturity selection to maintain viability will be weak in view of its high metabolic cost and the low contribution to Darwinian fitness which such an investment in maintenance confers (Hayflick, 2007a; Demetrius, 2013). The age of reproductive maturity thus represents a critical point in the vulnerability of an organism to age-related diseases. Up to this age, the random age-related defects in the energy producing organelles will be repaired and hence the incidence of metabolic diseases will be rare. After the age of reproductive maturity, defects will not be repaired. They will persist with highly cumulative deleterious effects on the metabolic integrity of the organism. Accordingly, the incidence of age-related diseases, such as Alzheimer's disease, will increase exponentially with age.
- *Inverse cancer comorbidity*—Biochemical and epidemiological studies indicate that the sporadic forms of cancer and age-related neurological disorders, such as Alzheimer's disease (AD), Parkinson's disease, (PD) and amyotrophic lateral sclerosis (ALS) are inversely comorbid. This notion refers to a lower than expected probability of disease occurring in individuals diagnosed with other medical disorders. The relation between cancer and AD is now well documented. Cancer survivors have a lower risk of AD than those with cancer. Prevalent AD is related to a reduced risk for cancer, whereas a history of cancer is associated with a reduced risk for AD (Roe et al., 2010; Driver et al., 2012; Tabares-Seisdedos and Rubenstein, 2013).

We will show that these three criteria are inconsistent with the amyloid hypothesis but concord with the predictions of the Inverse Warburg model. This observation will be invoked as the rationale for abandoning the amyloid hypothesis, and for advocating the processes involving metabolic reprogramming and natural selection as the effective model for the origin and progression of sporadic forms of AD.

The article is organized as follows: Section Bioenergetics outlines in quantitative terms the two principal modes of energy production, oxidative phosphorylation and glycolysis, involved in brain metabolism. Here we also describe how energetics in the brain is based on the integration of these two modes of energy production. Section The Origin and Progression of AD describes the bioenergetic model of the origin of AD. We apply the theory to distinguish between what we describe as normal aging and pathological aging, and the transition from normality to pathogenesis. Within this conceptual framework, AD is a derivative of pathological aging. Section Sporadic Forms of AD—Genetic and Metabolic contrasts the Amyloid Cascade model with the Energetic selection model. Empirical support for the existence of the Inverse Warburg effect is summarized in Section The Energetic Selection Model: Empirical Considerations. The notion that the up-regulation of OxPhos activity in neurons induces the sequence of events that could ultimately lead to AD has important implications for diagnostic and therapeutic strategies. These strategies are discussed in Section Therapeutic Implications: Suppressing the Inverse Warburg Effect.

## Bioenergetics

Our model for the origin of sporadic forms of AD postulates that energy and age are the two critical elements which drive the metabolic processes which culminate in neuronal loss, the histopathological hallmark of AD. Oxidative phosphorylation (OxPhos) and substrate phosphorylation are the principal mechanisms of energy production in cells. An understanding of the activity of these two modes of energy production in ensuring that the aging brain has sufficient energy is thus central in any study of the origin of neurological diseases

### Oxidative phosphorylation and substrate phosphorylation

The main energy currency in living organisms is ATP which must be continually available to maintain cell viability. The energy is derived from two types of processes: OxPhos, which provides about 88% of the total energy in most eukaryotic cells, including neurons, and substrate phosphorylation (mainly glycolysis) which contributes the remaining 12%. Oxidative phosphorylation occurs within the mitochondria. Electrons are transferred to oxygen via a series of redox reactions to generate water. In this process, protons are pumped from the matrix across the mitochondrial inner membrane through a set of respiratory complexes. When protons return to the mitochondrial matrix down their electrochemical gradient, ATP is synthesized via the enzyme ATP synthase. Energy production in this context is electrochemical. The rate of energy production is determined by the conductance of the biomembrane and the electromotive potential across the membrane (Nicholls and Ferguson, 2002).

**Figure 1:**
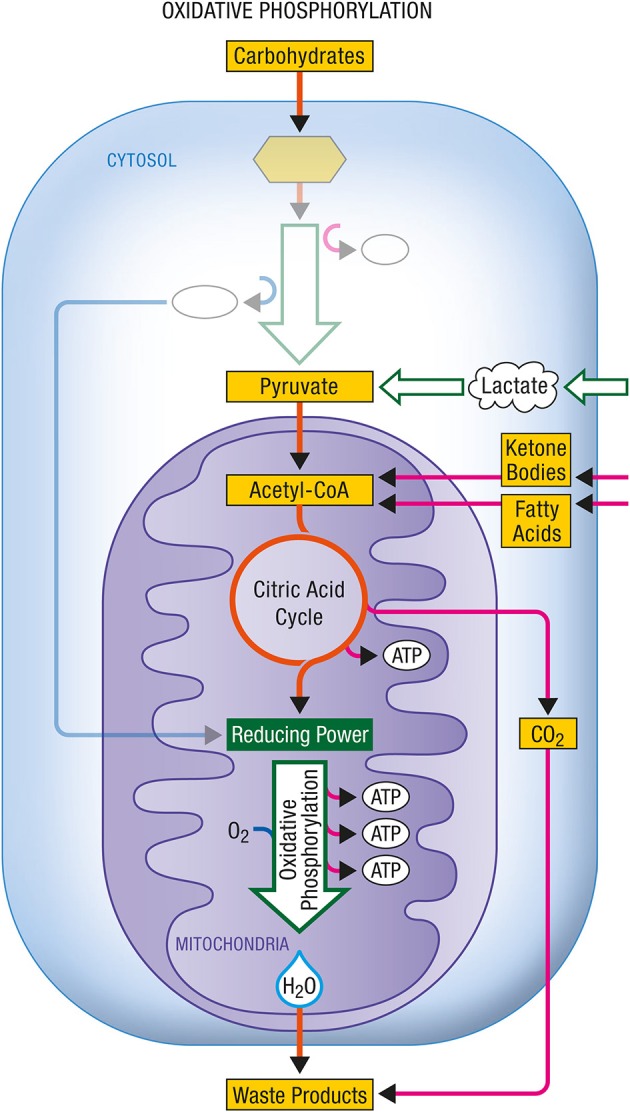
**Oxidative phosphorylation**. Coupled to the citric acid cycle, oxidative phophorylation allows the oxidative degradation and energy production from various energy substrates which include carbohydrates (in particular glucose after its conversion into pyruvate via glycolysis), lactate, ketone bodies or fatty acids. Both citric acid cycle and oxidative phosphorylation take place within mitochondria and give rise to carbon dioxide (CO_2_) and water (H_2_O) as waste products. ATP, Adenosine triphosphate.

**Figure 2:**
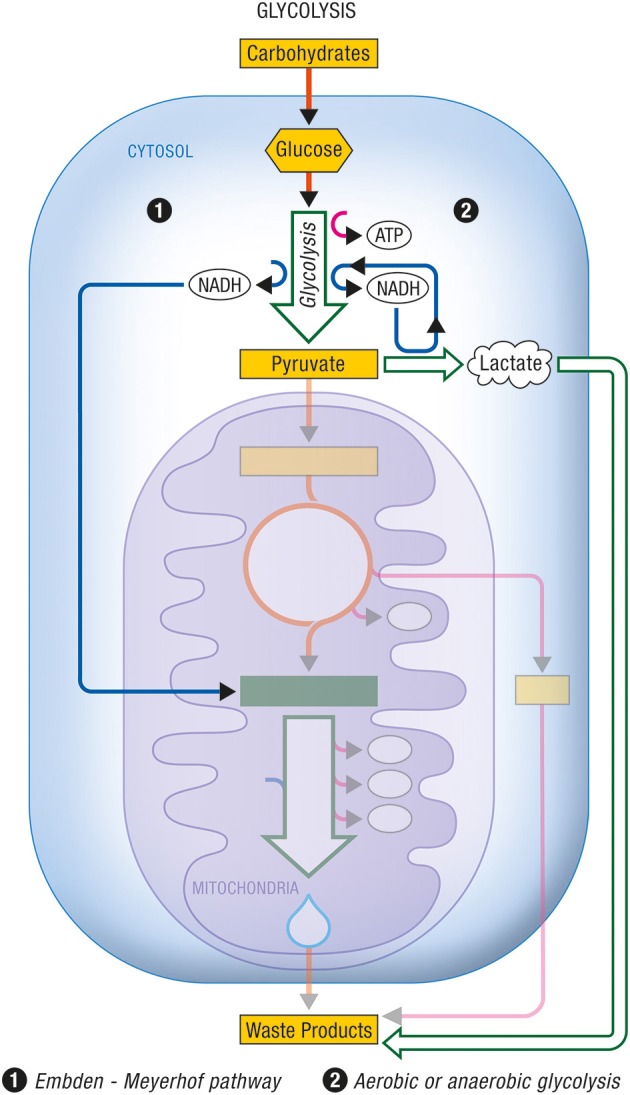
**Glycolysis**. Glycolysis is the non-oxidative part of the metabolic pathway that allows the use of carbohydrates by eukaryotic cells. (1) The Embden-Meyerhof pathway refers to the non-oxidative conversion of glucose (a major carbohydrate) into pyruvate prior to its entry into the citric acid cycle and its subsequent oxidation. Cytosolic NADH is reoxidized into NAD^+^ through specific mitochondrial shuttles. (2) Anaerobic glycolysis represents the conversion of glucose into lactate as an end product under conditions of limited oxygen availability. Aerobic glycolysis describes the same metabolic production of lactate as end product from glucose despite adequate oxygen availability to normally carry on complete oxidation of pyruvate. In these cases, cytosolic NADH is reoxidized within the cytosol by the conversion of pyruvate into lactate via the enzyme lactate dehydrogenase. ATP, Adenosine triphosphate; NADH, nicotinamide adenine dinucleotide (reduced form).

Glycolysis occurs within the cytosol. The glycolytic enzymes occur in relatively stable multienzyme complexes with metabolites passed on from one active site to the next without exchanging with the bulk cytoplasm. Energy production in this case is chemical. The rate of energy production is now determined by the activity of the glycolytic enzymes in the cytosol. In Figures 1, 2 are described the generation process of biological energy in the case of oxidative phosphorylation and glycolysis, respectively.

Quantum metabolism (Demetrius et al., 2010), an analytic theory of bioenergetics, provides a framework for deriving expressions for the metabolic rate of cells, and the dependence of this rate on the mechanism of energy transduction, OxPhos, or glycolysis. A cornerstone of the theory is the allometric relation between metabolic rate, P, and cell size, *W.* This is given by:

(2.1) $$P=\alpha W^{d/d+1}$$

Here, the integer *d* in the scaling exponent satisfies the relation, *1* < *d* < ∞.

The proportionality constant α is contingent on the mechanism of energy transduction, OxPhos or glycolysis. In the case of OxPhos, the proportionality constant α is determined by the proton gradient across the mitochondrial membrane. This quantity is largely determined by the phospholipid composition of the membrane. We have α = Δp, where:

(2.2) Δp=Δψ−cΔpH,

Here Δψ denotes the electrochemical gradient and ΔpH, the *pH* difference, and *c* a numerical constant. In the case of glycolysis, α = k where:

(2.3) k=c exp[−E/RT]

**Figure 3:**
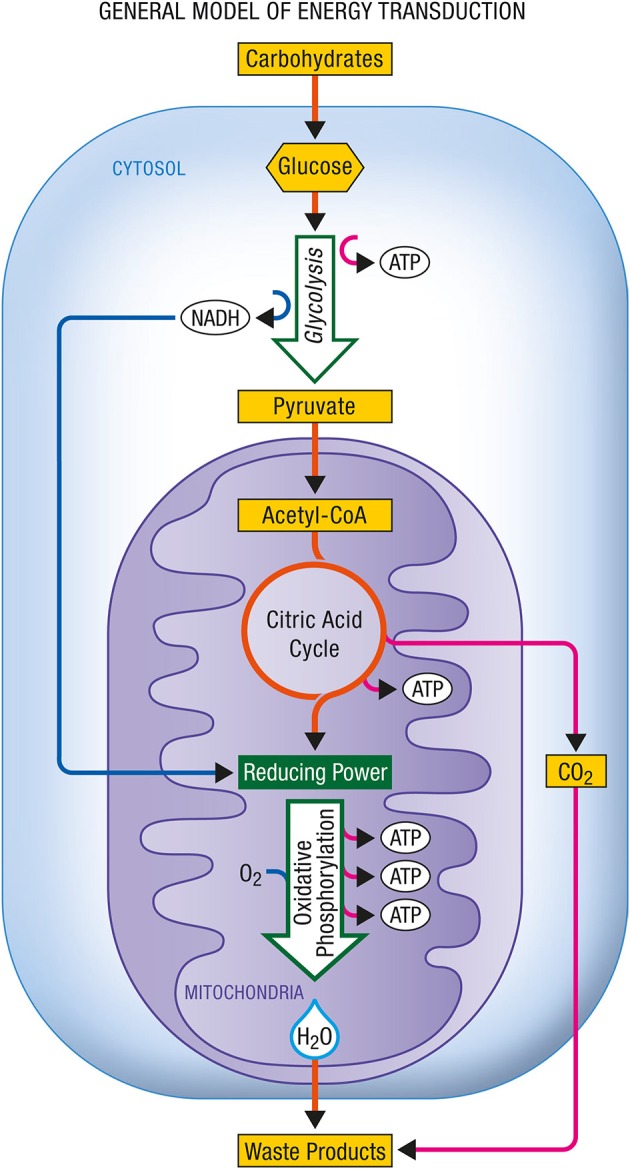
**General model of energy transduction**. In eukaryotic cells that rely essentially on carbohydrates for their energy production, the conjonction of glycolysis, the citric acid cycle and oxidative phosphorylation is responsible for the production of energy as ATP. Upon complete oxidation of energy substrates, both carbon dioxide (CO_2_) and water (H_2_O) are produced. ATP, Adenosine triphosphate; NADH, nicotinamide adenine dinucleotide (reduced form).

Figure 3 shows the energy production associated with the conversion of glucose to pyruvate and with the complete oxidation of pyruvate to carbon dioxide and water as it occurs in aerobic cells relying on glucose as their main energy substrate.

Glycolysis is a primitive way for anaerobic and facultative cells to obtain energy. The primitive nature of the process is indicated by the fact that the enzymes which catalyze the sequence of reactions exist free in solution in the cytosol. Oxidative phosphorylation is an emergent property of energy production. The enzymes which catalyze the reactions of the respiratory chain are located in the inner membrane of the mitochondria, a complex molecular fabric of lipid and protein molecules. In sharp contrast to the enzymes involved in glycolysis, the enzymes of the electron transport chain are located next to each other in the membrane in the exact sequence in which they interact (Lehninger, 1965; Harold, 2001).

The degree of organization of the enzymes in the cytosol and the respiratory chain enzymes imposes constraints on the metabolic rate generated by glycolysis and oxidative phosphorylation, respectively. In the case of glycolysis the metabolic rate will be determined primarily by the kinetic activity of the enzymes, as shown by Equation (2.2). However, the rate of energy production, in the case of oxidative phosphorylation, will depend on quantities such as the proton motive force, and the phospholipid composition of the membrane, as indicated in Equation (2.1).

### Neuroenergetics

The brain has important energy needs compared to other organs. To satisfy them, it needs a constant supply of both oxygen and nutrients. This is provided by an important and sustained cerebral blood flow. Glucose represents by far the main energy substrate for the adult brain. Most of the energy necessary to support brain activity is generated by the metabolism and oxidation of glucose via the tricarboxylic acid cycle coupled to oxidative phosphorylation in mitochondria (Magistretti, 2011). Changes in activity within specific brain areas lead to localized enhancement in blood flow as well as in glucose utilization. Failure to provide either oxygen and/or glucose in adequate amounts even to a small brain area can have rapid and dramatic consequences, e.g., ischemic neuronal death, indicating the importance of energetics for proper brain function.

The contribution by the main cell types (neurons and glia) constituting the brain to cerebral energy consumption has been estimated (Attwell and Laughlin, 2001; Hyder et al., 2006). At rest, the greatest portion of energy expenditure (~87%) is attributed to neurons while glial cells (with astrocytes being the dominant type) account for only a small fraction (~13%). Most of the energy consumed by neurons is dedicated to re-establish the ion gradients (via activity of the Na^+^, K^+^-ATPase) following their depolarization, mostly by excitatory post-synaptic potentials with a smaller contribution by action potentials (Alle et al., 2009). Neurons are highly oxidative cells and contain numerous mitochondria. For a long time, it was considered that direct glucose utilization and oxidation by mitochondria should be the main source of ATP for neurons both at rest and during periods of activity. Recent findings however questioned this view. It was found that neurons exhibit a low level of expression of PFKFB3, a critical enzyme for the regulation of glycolysis (Herrero-Mendez et al., 2009). The consequence is that neurons have a limited capacity to upregulate glycolysis to face enhanced energy demands. In contrast, astrocytes express high levels of PFKFB3 but also low levels of an important component of the malate-aspartate shuttle, the aspartate-glutamate carrier aralar, which is important for shuttling cytosolic NADH within mitochondria and promoting glucose-derived pyruvate oxidation instead of lactate formation (Ramos et al., 2003). Moreover, astrocytes exhibit low levels of pyruvate dehydrogenase activity (Halim et al., 2010). Modeling studies have shown that these characteristics explain why neurons are essentially oxidative cells while astrocytes have such a high glycolytic capacity (Neves et al., 2012). Furthermore, neurons have a low expression of the glyoxylases 1 and 2, two enzymes that are critical for metabolizing methylglyoxal a toxic byproduct of glycolysis (Bélanger et al., 2011). In contrast astrocytes can very effectively metabolize the glycolysis-induced formation of methylglyoxal, and in fact protect neurons against its toxicity (Bélanger et al., 2011). In parallel, it was shown both *ex vivo* and *in vivo* that glucose utilization is lower than predicted by energy expenditures in neurons while it is the opposite for astrocytes (Chuquet et al., 2010; Jakoby et al., 2014). These observations have several important implications:

1. To face higher energy demands, neurons cannot up-regulate glycolysis and must rely entirely on an enhancement of oxidative phosphorylation.
2. To face higher energy demands, neurons cannot oxidize more glucose-derived pyruvate (as their glycolytic capacity is limited) and must have access to another oxidative substrate (most likely lactate).
3. Astrocytes have constitutively high glycolytic rate giving rise to elevated glucose consumption and lactate production as opposed to neurons.

**Figure 4:**
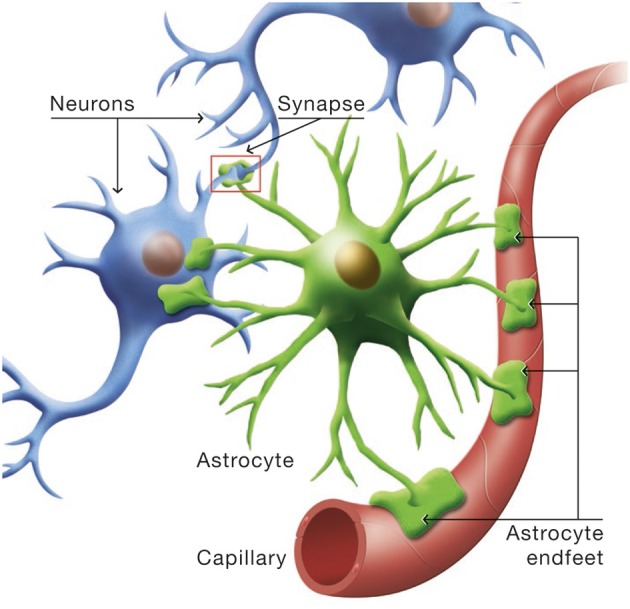
**Cytoarchitectural relationships of astrocytes**. Astrocytes (in green) are in contact with cerebral blood vessels such as capillaries (in red) through processes called endfeet. Moreover, astrocytes have other processes in the vicinity of neurons (in blue) that also ensheath synapses.

**Figure 5:**
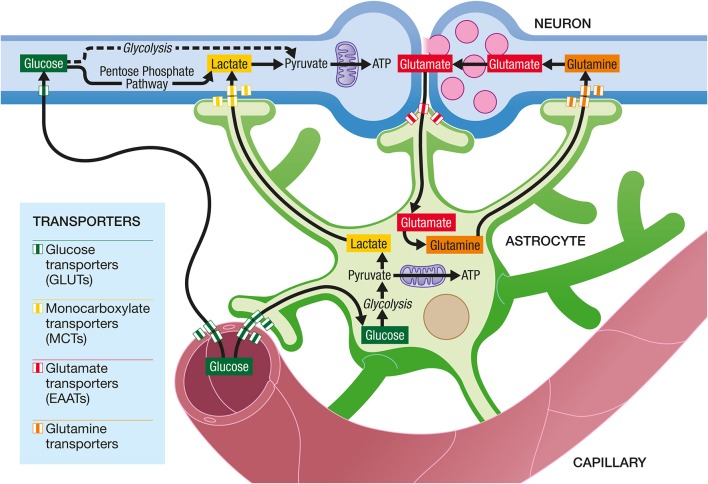
**The Astrocyte-Neuron Lactate Shuttle Hypothesis of activity-dependent regulation of neuronal energy substrate supply by astrocytes**. At excitatory synapses, glutamate is released in the synaptic cleft upon activation. Its action on post-synaptic receptors is terminated by its reuptake in astrocytes through high affinity, glial-specific glutamate transporters (EAATs). Glutamate is converted into glutamine and released by astrocytes via a specific glial glutamine transporter. Glutamine will be taken up by neurons from the extracellular space via a neuron-specific glutamine transporter and glutamine will be converted to glutamate before being accumulated in synaptic vesicles. In parallel, glutamate uptake into astrocytes will trigger an increase in glycolysis, with an enhancement of glucose uptake from the blood circulation into astrocytes via specific glucose transporters (GLUTs) on both endothelial cells and astrocytes. Lactate produced by astrocytes will be released in the extracellular space. To face their increased energy demands following synaptic activation, neurons will take up more lactate and oxidize it to produce more ATP. Neurons also take up glucose from the circulation via a specific glucose transporter. Part of the glucose can be metabolized through glycolysis and then oxidized in mitochondria to provide ATP. However, a significant amount of glucose is metabolized in neurons through the Pentose Phosphate Pathway (PPP) in order to regenerate NADPH necessary as cofactor for enzymes involved in antioxidant defenses.

Astrocytes occupy a strategic position as they are often interposed between blood vessels (the source of the main brain energy substrate glucose) and neurons (Figure 4). Indeed, they possess specific structures called end-feet that come in contact with cerebral blood vessels and cover almost entirely their surface. They also have other processes that ensheath synapses, allowing them to play important roles in relation with synaptic activity, e.g., recycling the neurotransmitter glutamate. Several years ago, it was proposed that lactate formed and released by astrocytes in an excitatory activity-dependent manner could provide an additional oxidative energy substrate for neurons (Pellerin and Magistretti, 1994). This model, known now as the Astrocyte-Neuron Lactate Shuttle (ANLS; Pellerin and Magistretti, 2012), describes a cellular and molecular mechanism (including lactate supply by astrocytes to neurons) that provide an explanation for this partial compartmentalization of glycolysis and oxidative phosphorylation in the central nervous system (Figure 5). It emphasizes also the importance of a metabolic cooperation between neurons and astrocytes to sustain neuronal activity.

Recent studies, reviewed in Zilberter et al. (2010), have indicated the importance of lactate as a cerebral oxidative energy substrate. The human brain can aerobically utilize lactate as an energy substrate by the tricarboxylic acid cycle. A significant and critical discovery is that the composition of the energy substrates utilized by the brain is contingent on age, energy demands and physiological conditions. During increased neuronal activity, significant changes in the concentrations of the energy substrates occur (Hu and Wilson, 1997; Pellerin et al., 2007). Glucose concentration is lowered and lactate concentration is increased, indicative of an enhanced activity of the astrocytes.

The Inverse Warburg hypothesis as it will be developed in the following sections requires only that the lactate produced by astrocytes is a source of energy for neurons. Although the biochemical details of how it is produced and transferred to neurons are not relevant to the Inverse Warburg hypothesis, the ANLS model provides an interesting conceptual framework to further explore the implications of an energetic dysfunction in Alzheimer's disease. For this reason, it has been integrated in the description of the processes encompassed by the Inverse Warburg hypothesis. However, it is important to make it clear that the Inverse Warburg hypothesis described herein does not rely on this model for its own validity, although it certainly raises its appeal.

## The origin and progression of AD

**Figure 6:**
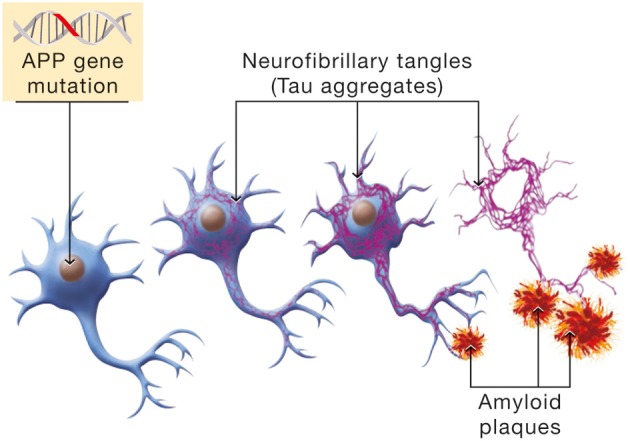
**Molecular mechanism involved in the β-amyloid cascade hypothesis of Alzheimer's disease**. Mutations in the Amyloid Precursor Protein (APP) gene will induce a cascade of events leading to neuronal cell death. Hyperphosphorylation of the Tau protein is one manifestation of the β-amyloid cascade which has for consequence the formation of NeuroFibrillary Tangles (NFTs, in purple). In parallel, APP mutations cause erroneous β-amyloid protein processing and β-amyloid deposition in senile plaques.

Epidemiological data distinguish between early onset and late onset AD. A small minority of individuals are afflicted with the early onset form of the disorder. This is an autosomal dominant form attributable to mutations in three genes, APP, presenilin 1 and presenilin 2. The expression of the disease follows a pattern of Mendelian inheritance. Hallmarks of the disease are the formation of neurofibrillary tangles (Tau aggregates and the deposition of amyloid plaques; see Figure 6).

The age of incidence is a random variable whose distribution has the form of a bell-shaped curve described by a minimum and maximum age of onset of 35 and 65 years, respectively, and a mean age of 50 years (Hendrie, 1998; Swerdlow, 2007).

The late onset form of the disease does not follow Mendelian inheritance, although it shows some degree of heritability. There exist a number of genetic risk factors for AD. However the major risk factor is age. The age of onset is about 70 years, and disease prevalence increases exponentially with age.

The symptoms of AD are initially very mild. They then slowly progress. The neuronal cell types show selective vulnerability. Neurodegeneration typically begins in the entorhinal cortex, and then spreads to the hippocampus and parietal regions of the neocortex, Hof and Morrison (2004). Both the early and late onset forms of the disease show similar histopathological changes.

The amyloid cascade hypothesis and the energetic selection model are two conceptual frameworks that have been invoked to explain the origin of sporadic forms of AD (Hardy and Selkoe, 2002; Hardy, 2009; Demetrius and Simon, 2012; Demetrius and Driver, 2013). Both models acknowledge the epidemiological fact that age is a primary risk factor for the disease. However, the effect of age on the dynamics of neurodegeneration is analyzed in quite distinct ways.

### The amyloid cascade model

**Figure 7:**
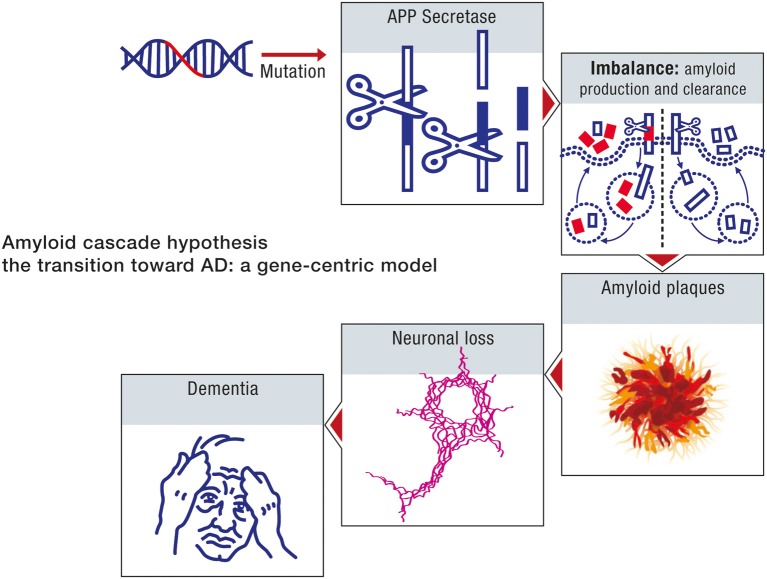
**Amyloid cascade hypothesis: a gene-centric model**. A mutation in the amyloid gene or one of the genes involved in amyloid processing (e.g., presenilin) will provoke an imbalance between amyloid production and clearance. As a consequence, amyloid deposition will take place, forming amyloid plaques, and neurofibrillary tangles will also appear. Neuronal death will ensue, leading progressively to dementia.

The amyloid cascade hypothesis is based on a neuron-centric characterization of the origin and development of the disease. The model essentially does not take into account the interaction between neurons and astrocytes in disease progression. The beta amyloid moieties, the presumed biochemical hallmarks of neurodegeneration, are assumed to be generated by abnormal processing of APP. Age in the context of this model is considered uniquely in terms of its influence on the toxicity of the peptide beta amyloid. In this model, age reflects the time it takes beta amyloid to attain concentrations which are sufficient to impair neuronal function. Accordingly, the sporadic form of the disease can be considered to be determined primarily by the net production rate of beta amyloid. Since this biochemical abnormality is assumed to be determined by aberrant processing of APP, the early onset and sporadic forms of the disease can be ascribed the same etiology. The sporadic form of AD is thus a consequence of genomic instability, primarily the result of mutations in the nuclear genome, and hence can be considered to be a genetic disease. The sequence of pathogenic events leading to AD, in accordance with the amyloid cascade hypothesis is shown in Figure 7.

### The inverse warburg hypothesis

**Figure 8:**
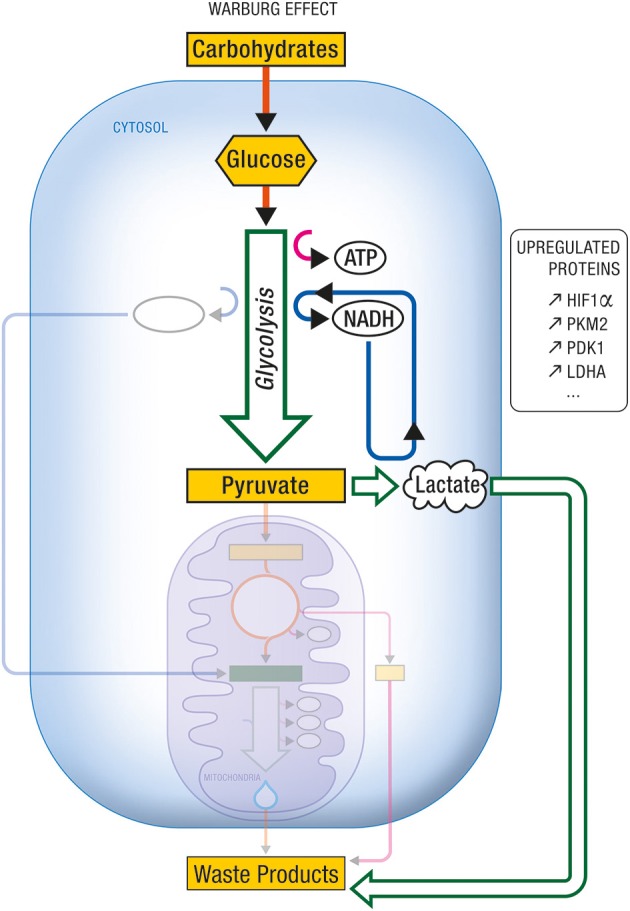
**Warburg effect**. Under certain conditions (e.g., tumorigenicity, proliferation), aerobic glycolysis becomes the predominant form of carbohydrate metabolism and energy production, at the expense of citric acid cycle and oxidative phosphorylation. One characteristic of this metabolic imbalance is the upregulation of several proteins and enzymes involved in glycolysis and/or in its regulation. Among others, they include the transcription factor HIF-1α, or the enzyme isoforms PKM2, PDK1, and LDHA. ATP, Adenosine triphosphate; HIF-1α, Hypoxia Inducible Factor-1α; LDHA, Lactate dehydrogenase A; NADH, nicotinamide adenine dinucleotide (reduced form); PDK1, Pyruvate dehydrogenase kinase 1; PKM2, Pyruvate kinase M2.

**Figure 9:**
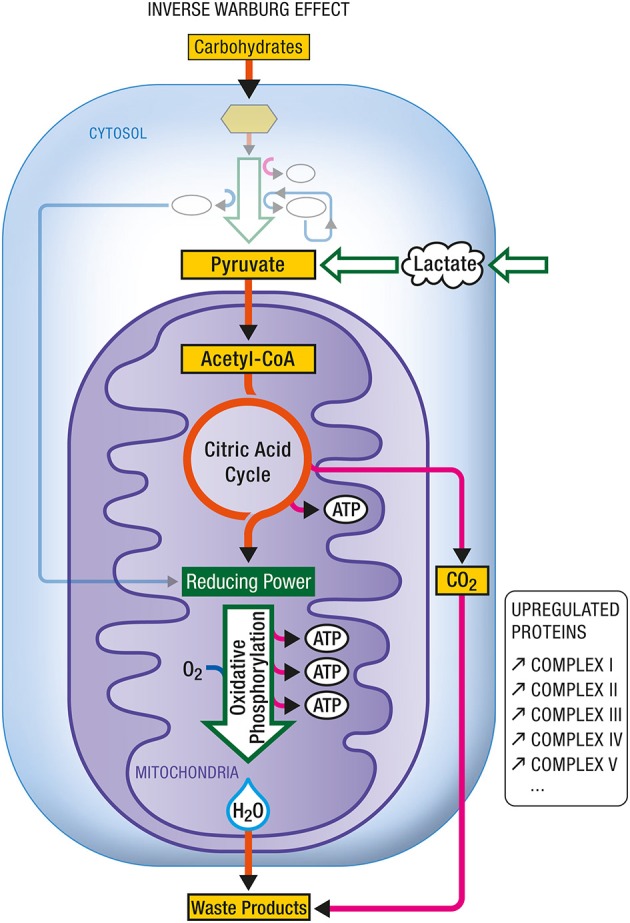
**Inverse Warburg effect**. In some instances (e.g., during aging), mitochondrial dysregulation leads to a dysproportionate upregulation of oxidative phosphorylation. In such case, an enhancement in the expression of all complexes (I–V) composing the respiratory chain has been documented. In addition to carbohydrate-derived pyruvate, cells become critically dependent on other oxidative substrate sources such as lactate-derived pyruvate. ATP, Adenosine triphosphate.

The neuroenergetic model is based on a neuron-astrocytic characterization of energy supply and demand in neuronal and glial cells. This model recognizes that brain energy metabolism involves both neurons and astrocytes (Pellerin and Magistretti, 1994, 2012). Both cell types utilize glucose as an energy source. In astrocytes, a significant proportion of glucose is metabolized aerobically to lactate which is released into the extracellular milieu. In neurons, glucose-derived and lactate-derived pyruvate is metabolized aerobically, oxidative phosphorylation being the predominant mode of energy production. Neurons are unable to increase energy production through glycolysis owing to the lack of activity of some glycolysis promoting enzymes (Bolanos et al., 2010). Hence when some of the mitochondria in neurons become impaired, the associated increased demand for energy is achieved by the action of two events. The first is the up-regulation of glycolysis in astrocytes, a metabolic reprogramming which results in increased production of lactate. The second is the up-regulation of OxPhos activity in neurons, which will use more lactate produced by astrocytes as an additional source of energy. The up-regulation of glycolysis and the up-regulation of OxPhos activity are two complementary modes of metabolic reprogramming. The first is called the Warburg effect (Figure 8), the second, the Inverse Warburg effect (Figure 9).

**Figure 10:**
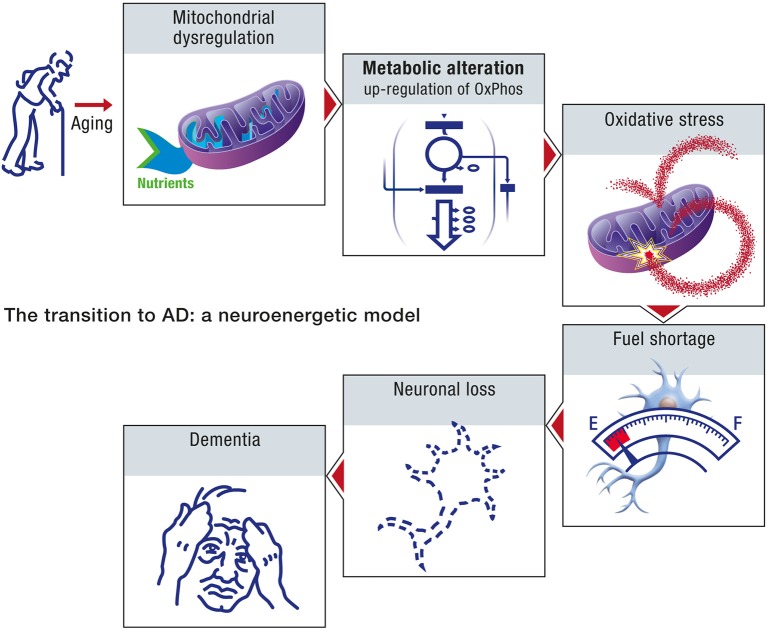
**The transition to AD: a neuroenergetic model**. As a consequence of aging, mitochondrial dysregulation occurs which will lead to an upregulation of oxidative phosphorylation as well as oxidative stress. Since upregulated mitochondrial oxidative activity will require more energy substrates to produce the same amount of energy, it will eventually lead to an energy shortage, especially for the other cells that have not undergone upregulated oxidative phosphorylation. Neurons that cannot compete for energy substrates and produce sufficient amount of energy will eventually die, leading progressively to dementia.

Age, in the context of this neuroenergetic model, is defined in terms of its effect on the aggregation dynamics of the cellular proteins, and the metabolic capacity of aged neurons. At the molecular level, the aging process is driven by an increase in molecular disorder and a concomitant decrease in the activity of the enzymatic processes (Hayflick, 2007a). Aging can result in the decline of chaperone and proteasome responses to protein aggregation and hence to amyloid formation. At the metabolic level, the aging process will induce a decrease in the efficiency with which neurons appropriate caloric energy and transform this energy into ATP. The neuroenergetic model posits that this reduced efficiency will trigger a cascade that involves the up-regulation of oxidative phosphorylation in neurons, oxidative stress and further damage to mitochondria, fuel shortage, neuronal loss and dementia (Figure 10).

The mitochondria in neurons are the energy producing organelles. This energy depends on the proper functioning of the metabolic network whose efficiency is contingent on the maintenance of the precise three dimensional structures of the biological molecules. The increase in molecular disorder which the aging process induces is a consequence of the intrinsic thermodynamic instability of these complex biomolecules (Hayflick, 2007b). Molecular disorder can be analytically described in terms of the concept *thermodynamic entropy*, denoted S. Thermodynamic entropy has both a statistical and a macroscopic representation. The statistical representation is due to Boltzmann and is given by:

S=k log W

The constant *k* is Boltzmann's constant. The quantity *W* is a measure of the number of ways that the molecules of a system can be arranged to realize the same total energy. In qualitative terms, *S* describes the extent to which the energy in the system is spread out over the various microscopic storage modes. The macroscopic representation, which is due to Clausius is given by:

(3.1) $$dS=\frac{dQ}{T}$$

Here *dS* denotes the change in entropy which occurs when an energy *dQ* is transferred as heat to the system. The quantity *T* denotes the temperature at which the transfer took place.

The Second Law of thermodynamics asserts that in isolated systems, the thermodynamic entropy will increase. We can exploit this principle to explain the inevitable decrease in the ability of complex biomolecules to maintain their three dimensional folded state, and hence their function and catalytic activity. The inherent, thermodynamic instability of protein molecules entail that conformational alterations and aggregation will occur, leading to misfolded structures (Chiti and Dobson, 2009).

The increase in thermodynamic entropy will also have an effect on molecular fidelity and hence an increase in the mutation rate of mitochondrial DNA (Hayflick, 2007a). These age-specific changes will also affect the kinetic activity of the various enzymes involved in reactions which transform the energy contained in substrates, like glucose and lactate, into energy which can be used to maintain the integrity of synaptic connections. The effect of these alterations in kinetic activity will be a decrease in the efficiency of the metabolic process—a condition which can be described in terms of a decrease in *evolutionary entropy* (Demetrius, 2013). Evolutionary entropy is a measure of dynamical organization of biological networks, that is systems which appropriate resources from the external environment and convert these resources into chemical energy. These networks include energy producing systems such as the glycolytic and oxidative phosphorylation systems in cells, demographic systems in which the individual elements are different stages in the individual life cycle. Evolutionary entropy describes the number of pathways of energy flow within a biological network. As is the case with thermodynamic entropy. Evolutionary entropy admits both statistical and macroscopic representations (Demetrius, 2013).

The statistical representation, an analog of the Boltzmann entropy, is given by:

$$\tilde{S}=\tilde{k}\log\tilde{W}$$

Here $\tilde{k}$ is a numerical constant. The quantity $\tilde{W}$ denotes the number of pathways of energy flow within the network.

Glycolytic and oxidative phosphorylation networks are described in terms of a small and a comparatively large number of pathways of energy flow, respectively. This entropy measure also describes the rate at which the system appropriates energy from the external environment, and converts this energy into cellular work.

The macroscopic representation of evolutionary entropy is:

(3.2) $$d\tilde{S}=-\tilde{T}d\tilde{P}$$

The relation given by Equation (3.2) describes the change in entropy *d*$\tilde{S}$ which occurs when a quantity of energy, as measured by the change in metabolic rate *d*$\tilde{P}$ is introduced to the system, which is processing energy with a cycle time or turnover time $\tilde{T}$.

Our model posits that the aging process will induce perturbations at both molecular and cellular levels. The changes at the molecular level are the result of an increase in thermodynamic entropy. These entropic changes are associated with an *increase* in molecular disorder, and will induce mutations in mitochondrial DNA and misfolding of various proteins. Amyloidosis, in the context of this model, is primarily a consequence of aging, and its bioenergetic signature, thermodynamic instability.

The changes at the cellular level are the result of a *decrease* in evolutionary entropy. These entropic changes will result in metabolic dysregulation, and a decrease in the energy production of neuronal cells.

The mitochondrial dysregulation which the aging process induces will have critical effects on the capacity of impaired neurons to maintain ATP levels which are necessary to ensure metabolic homeostasis. The Inverse Warburg hypothesis postulates the following cascade of events as the adaptive response to the bioenergetic stress caused by mitochondrial deficits.

**Figure 11:**
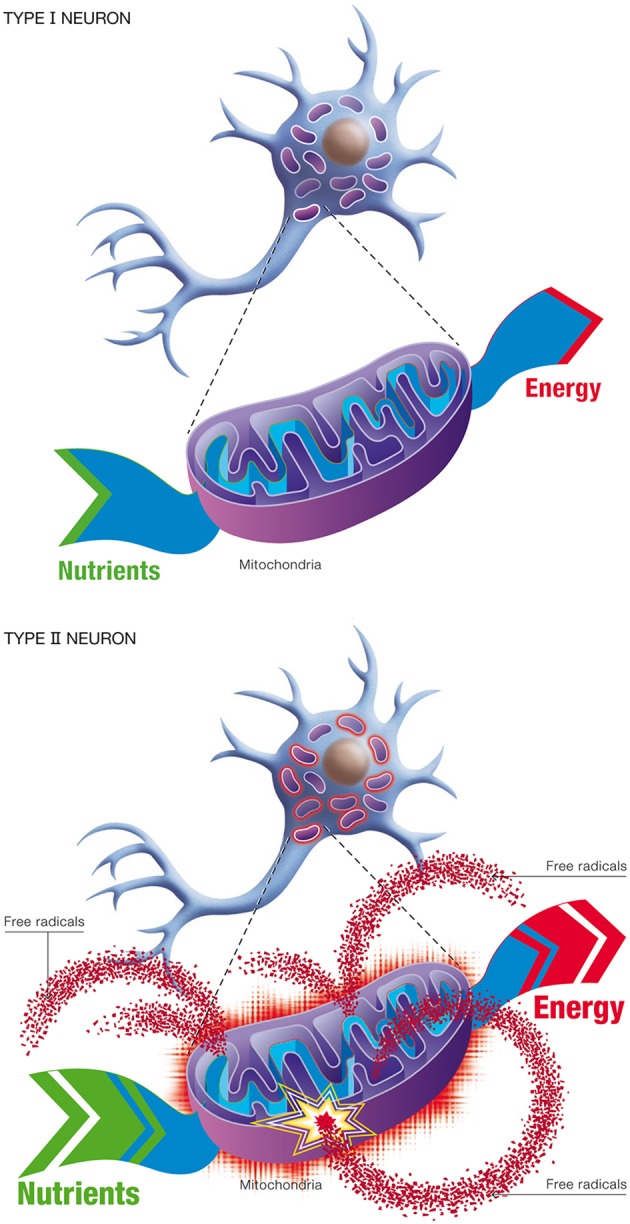
**Changes in neuronal energetic efficiency during normal aging**. Neurons rely heavily on oxidative metabolism for obtaining their energy from nutrients. This process takes place in mitochondria. Healthy neurons with maximal energetic efficiency are designated type I neurons. During normal aging, mitochondrial instability and dysfunction appears, notably caused by the reduction of antioxidant defenses and the formation of oxygen free radicals. As a consequence, certains neurons will undergo a reduction in energetic efficiency and higher metabolic rate, requiring more nutrients to sustain their energy needs. These neurons are called type II neurons.

- *Metabolic alteration.* This pertains to the Inverse Warburg effect, the up-regulation of OxPhos activity in the impaired neurons to compensate for the diminished efficiency of energy production.
  This mode of metabolic reprogramming recognizes the inherent complexity of mitochondrial dynamics. The large requirements of neuronal energy is achieved by the fission and fusion of mitochondria to generate smaller or elongated organelles (Van Laar and Berman, 2013). In impaired neurons, a small subset of mitochondria may become defective with age, while the other organelles remain intact. The metabolic alteration we postulate involves an increase in OxPhos activity in the intact mitochondria, and a redistribution of these mitochondria to sites which require high energy production. Such movement of mitochondria in neurons has already been documented (Chang and Reynolds, 2006).
- *Natural selection.* This process describes competition for lactate (generated by astrocytes) as the preferred oxidative substrate between intact neurons (Type I), with normal OxPhos activity, and impaired neurons (Type II), with up-regulated OxPhos activity (Figure 11). The outcome of competition between the two classes of neurons is described by the *entropic selection principle*.

According to the entropic selection principle the outcome of competition between an incumbent population of cells and a variant population is predicted by evolutionary entropy, and is contingent primarily on resource abundance and resource composition. The evolutionary entropy of the neuron is defined in terms of the number of pathways of energy flow in the oxidative phosphorylation network. Neurons with up-regulated OxPhos activity have a high evolutionary entropy, when compared with neurons with normal OxPhos activity (Demetrius and Simon, 2012, 2013).

The microenvironment generated by the metabolic activity of astrocytes will be characterized by lactate, Astrocytes, like neurons, also age. Accordingly, lactate production in older individuals will be limited. This limited resource constraint, according to the selection principle, entails that neurons with up-regulated OxPhos activity (the impaired neurons), will outcompete neurons with normal OxPhos activity (the intact neurons). This means that the impaired neurons will have a selective advantage and increase in frequency.

The integrative action of metabolic reprogramming and natural selection on the neuronal population will be a gradual change in the distribution of intact and impaired neurons, resulting in a decrease in frequency of the normal cells, and an increase in frequency of the dysfunctional cells.

### Normal aging and pathological aging

The cascade of events generated by the three processes, mitochondrial dysfunction, metabolic alteration, and natural selection, can be more clearly delineated by appealing to concepts drawn from dynamical systems theory to distinguish between two gerontological stages, normal and pathological aging (Demetrius and Simon, 2013).

Normal aging is characterized by the incidence of intact and dysfunctional neurons in a quasi-stable equilibrium state. Impaired or dysfunctional neurons are defined by changes at the molecular and cellular levels. The changes at the molecular level involve an accumulation of amyloid plaques—a result of misfolded proteins. The alteration at the cellular level will reflect the effects of diminished energy production.

The quasi-stable state which defines the distribution of relatively intact neurons and impaired neurons will be maintained by various response mechanisms which have evolved to preserve cognitive function across an entire life span. These mechanisms include:

(i) *The action of anti-oxidant enzymes*.

These enzymes mitigate the deleterious effects caused by the excessive production of reactive oxygen species (ROS) generated by neurons with up-regulated OxPhos activity.

(ii) *The action of signal transduction enzymes.*

These enzymes repair defects in cell-cell signaling in neurons—an impairment which will occur when there is a significant decline in energy production due to impaired efficiency in enzymes in the electron transport chain.

(iii) *The activation of neuroprotective genes.*

The repressor element 1-silencing transcription factor (REST) is a universal property of normal aging in human cortical and hippocampal neurons (Lu et al., 2014). REST represses genes that promote neuronal loss and induces the expression of stress response genes.

Pathological aging is induced by a disruption of the quasi-stable state involving intact and impaired neurons. This dysregulation of the equilibrium condition will be generated by various insults which will impair the activity of the mechanisms which have evolved to preserve cognitive function and protect against neuronal loss in the aging brain. The morphological signature of pathological aging is a decreased synaptic density and loss of synaptic function. The cellular hallmarks are neuronal loss and loss of dendritic branching. A comparison between the features of normal and pathological aging is described in Table 1.

**Table 1:** **Comparison: normal and pathological aging**.

**Comparison**	**Normal aging**	**Pathological aging**
Clinical	Mild defects in short term memory	Severe defects in short term and long term memory
Molecular	Mild accumulation of amyloid plaques	Severe and diffuse accumulation of amyloid plaques
Cellular	Impaired synaptic function	Neuronal loss and synaptic loss, loss of dendritic branches

According to the energetic selection model for AD, neuronal loss is the histopathological signature of the disease following the collapse in the action of the various neuroprotective mechanisms that enhance neuronal viability. A factor which plays a central role in the transition toward pathological aging is oxidative stress. Under normal conditions, neurons never enter the cell cycle and once mature, they no longer have the capacity to complete mitosis. Oxidative stress is one of the factors which leads to abnormal cell cycle entry and ultimately neuronal loss.

## Sporadic forms of AD—genetic and metabolic

The peptide beta amyloid is constantly produced in the brain of young and old people. Extracellular beta amyloid levels rise during the day and fall at night (Huang et al., 2012). In the healthy brain, beta amyloid production and clearance is tightly regulated. In the unhealthy brain, the production and clearance is dysregulated. These considerations are of critical importance in both the amyloid hypothesis and the neuroenergetic model. However, the role which beta amyloid plays in the depictions of sporadic forms of AD are quite distinct and derive from different assumptions regarding the origin and the progression of the disease.

The amyloid cascade model is a neuron-centric analysis of disease origin and progression. The argument considers the progression of the disease in an energetically closed system which ignores the interaction of neurons with astrocytes. According to this model, neuronal loss is due to the formation of senile plaques generated by the loss of homeostatic regulation of beta amyloid.

The Inverse Warburg hypothesis is a neuron-astrocytic depiction of the transition to AD in which the mitochondria occupies the central and fundamental role. The model recognizes that brain energy metabolism involves both neurons and astrocytes and their interaction. The sporadic forms of AD, according to this model are driven by the aging process which acts on the mitochondria, which are vulnerable organelles because of their high mutation rates.

The notion that mitochondrial dysfunction is a key factor in age-associated neurodegenerative diseases has been articulated in several publications (Blass, 2000; Beal, 2005; Wallace, 2005; Swerdlow, 2007; Reddy, 2008; Yao and Brinton, 2011). These various studies have emphasized the role of age and energy in a variety of neurodegenerative diseases.

The Inverse Warburg hypothesis we have described builds on these empirical observations. The novelty of the energetic selection model as it pertains to the origin of age-related neurodegenerative diseases, rests on three critical factors, namely:

- The analytic integration of mitochondrial energetics in neurons with the cytosolic energetics in astrocytes—the *ANLS shuttle hypothesis* (Pellerin and Magistretti, 1994). It is noteworthy that the ANLS concept has been related to a similar phenomenon observed in the cancer field (known as the *Reverse Warburg effect*) whereby a support cell exhibits a high glycolytic activity in order to fuel a more oxidative neighbor cell (Pavlides et al., 2010).
- The promulgation of the concept of metabolic reprogramming (i.e., increased oxidative phosphorylation) as a compensatory mechanism to meet the energetic demands induced by mitochondrial dysfunction in neurons—the *Inverse Warburg effect* (Demetrius and Simon, 2012).
- The phenomenon of natural selection—competition between intact and impaired neurons for alternative fuel sources—the entropic selection principle (Demetrius, 1997; Demetrius and Driver, 2013).

It is of some interest to note that the astrocyte-neuron lactate shuttle (Pellerin and Magistretti, 1994) which is postulated here to regulate energy transduction in Alzheimer's disease is analogous to what we will describe as the stromal-epithelial lactate shuttle in oncology (see Pavlides et al., 2010). Both bioenergetic processes involve a system whereby support cells exhibit high glycolytic activity in order to fuel oxidative neighbor cells. In brain, the support cells are the astrocytes; in tumors, the support cells are stromal fibroblasts. The phenomenon of metabolic alteration as an adaptive strategy is observed in both neurodegeneration and tumorigenesis. Mitotic cells such as tumors can utilize both glycolysis and oxidative phosphorylation as means of energy production. Hence metabolic shifts to glycolysis or OxPhos activity are both viable adaptive options. The up-regulation of glycolysis in cancer cells is called the Warburg effect. The up-regulation of OxPhos activity, the *reverse* Warburg effect.

Post-mitotic cells such as neurons lack the capacity to make a compensatory metabolic shift to glycolysis due to the weak activity of the key glycolytic enzymes. Accordingly, a metabolic shift to up-regulate oxidative phosphorylation is the only viable adaptive strategy. We have called this metabolic alteration, the *Inverse Warburg* effect. The reverse and inverse Warburg effects are distinct phenomena, the first referring to mitotic cells, such as cancer; the second to post-mitotic cells such as neurons.

### Amyloid cascade hypothesis and the inverse warburg hypothesis: a contrast

The amyloid hypothesis is a neuron-centric argument. This model does not explicitly consider the interaction between neurons and the neuronal microenvironment. The Inverse Warburg hypothesis is a neuron-astrocytic model which takes into account the fact that astrocytes provide the energy source to maintain neuronal viability. We will contrast these two competing hypotheses by assessing their capacity to accomodate and explain three distinct hallmarks of AD. These disease hallmarks can be described as follows:

1. *Selective vulnerability:* Neuronal populations are characterized by differential sensitivity to stress that cause cell injury and death. Hippocampal and frontal lobe pyramidal neurons are highly vulnerable cell types, whereas dentate gyrus granule neurons and cortical interneurons are resistant to injury (Hof and Morrison, 2004; Mattson and Magnus, 2006).
2. *Age-dependence:* The disease has an age of onset of about 70 and the age-specific incidence rate increases exponentially with age (Hendrie, 1998; Swerdlow, 2007).
3. *Comorbidity:* There exists an inverse comorbidity between cancer and Alzheimer's disease. People with certain sporadic forms of cancer are protected against AD, whereas people without a history of cancer have a high risk of AD (Driver et al., 2012).

These three conditions represent critical biochemical and epidemiological aspects of sporadic forms of AD. Consequently, any model that purports to provide a mechanism for the origin and progression of the disease should be consistent with these biochemical and epidemiological hallmarks.

We will now contrast the explanatory power of the amyloid cascade hypothesis and the Inverse Warburg hypothesis by assessing the extent to which each model is consistent with the three properties: selective neuronal vulnerability, age-specific incidence rate and inverse cancer comorbidity.

(i) *Selective neuronal vulnerability*

The differences in the vulnerability of neurons to AD derive essentially from differences in the demand for energy to maintain their viability and to perform their function.

According to the Amyloid cascade hypothesis, neuronal loss is due to the toxicity of beta amyloid. However, since beta-amyloid derives from processing of the APP gene, which will be the same in each cell type under the amyloid cascade model, the vulnerability of each neuronal type should be essentially random and non-selective. This prediction of the amyloid hypothesis, however, is inconsistent with the selective neuronal vulnerability.

Selective vulnerability, however, is consistent with the energetic selection model. The main tenet of this model, the up-regulation of OxPhos activity as a compensatory response to impaired energy production, underscores its bioenergetic basis. The vulnerability of neurons depends on their energy demands in times of stress. Neurons providing the projection from entorhinal cortex to dentate gyrus and pyramidal cells have stronger energy demands, and are therefore most vulnerable (Hof and Morrison, 2004). Selective differences in neuronal vulnerability are thus an immediate derivative of the energetic selection model.

(ii) *Age-specific incidence rates*

**Figure 12:**
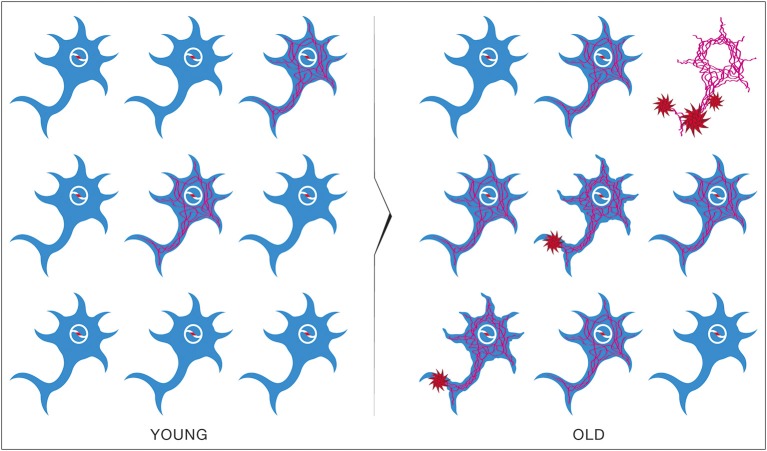
**Brain pathological evolution to Alzheimer's disease with age according to the β-amyloid cascade hypothesis**. Amyloid Precursor Protein (APP) gene mutations present in all neurons will cause the appearance in the most vulnerable neurons of NeuroFibrillary Tangles (NFT) in asymptomatic patients (YOUNG). With time, β-amyloid plaques will form and some neurons will eventually die (OLD). Progressively, more and more neurons will enter this process leading to the signs of cognitive decline characteristic of Alzheimer's disease.

The amyloid hypothesis assumes that the sporadic form of AD is primarily a *genetic* disease initiated by mutations in the nuclear genome. Age in this model is considered a measure of the time required for biochemical abnormalities to attain certain toxic concentrations (Figure 12). The age-incidence rate of the disease will therefore be determined by the genetic constitution of the population and the environmental factors. The effects of these processes on disease expression can be considered independent and additive. Consequently, the age-specific incidence rate of diseases subject to these constraints will be bell-shaped.

The Inverse Warburg hypothesis assumes that the sporadic form of AD is primarily a *metabolic* disease initiated by mitochondrial dysfunction. Age, in the framework of this model, determines pathogenesis by means of an increase in molecular disorder, a loss of molecular fidelity, and a decrease in efficiency of the metabolic processes. These effects are interdependent, and the intensity of their action on neuronal dynamics will be cumulative. Accordingly, the age-specific incidence rate of the disease will increase exponentially with age.

**Figure 13:**
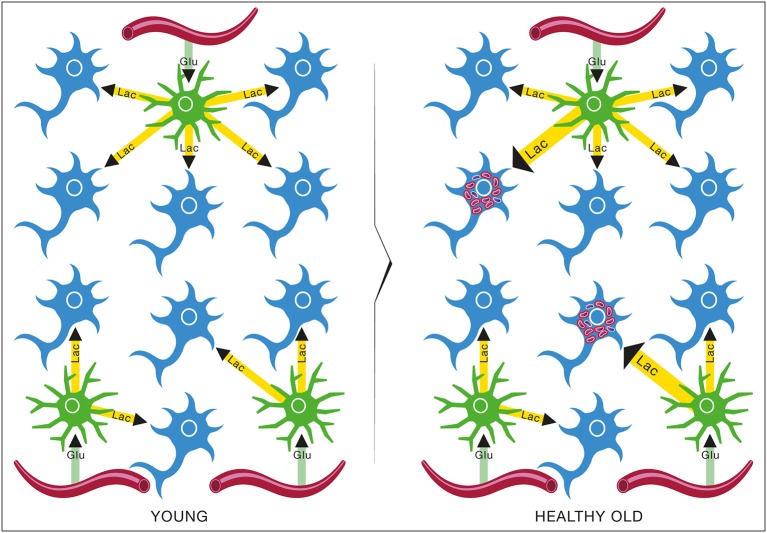
**Brain metabolic changes during normal aging**. Brain of young individuals is composed of normal type I neurons which are fed with lactate produced by astrocytes from glucose provided by the circulation (YOUNG). In normal aging, a few abnormal type II neurons with mitochondrial instability and dysfunction will appear but the increase in energy demands caused by these neurons can be met by the lactate production of astrocytes (HEALTHY OLD). Glu, Glucose; Lac, Lactate.

**Figure 14:**
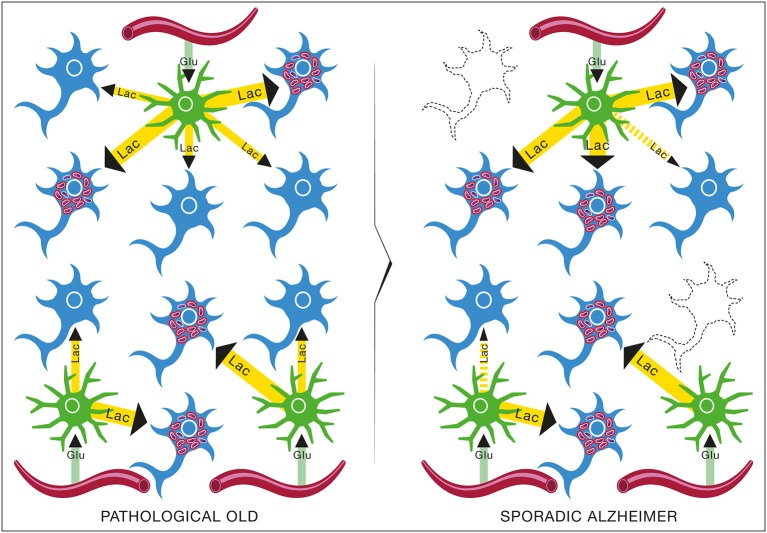
**Brain metabolic changes during pathological aging leading to Alzheimer's disease according to the Inverse Warburg hypothesis**. In pathological aging, the number of type II neurons with mitochondrial instability and dysfunction increases importantly, requiring a more important share of the lactate produced by astrocytes to sustain their energy needs (PATHOLOGICAL OLD). With time, as the number of type II neurons continue to rise, normal type I neurons do not receive enough lactate to support them, leading to damage and conversion as type II neuron and/or cell death (SPORADIC ALZHEIMER). Glu, Glucose; Lac, Lactate.

The epidemiological studies of the age-incidence rate of AD indicate an exponential increase (Hendrie, 1998). This fact does not support the neuron-centric model of AD. However, the empirical observations are consistent with the energetic selection model which considers AD as a metabolic disease. In the case of normal aging, despite the appearance of neurons with dysfunctional mitochondria, the system remains in an equilibrium state (Figure 13). This is due to the neuroprotective mechanisms activated to preserve homeostasis (Described in section The Origin and Progression of AD). In contrast, pathological aging results from an imbalance in these processes, accelerating the appearance of mitochondrial dysfunction. When this occurs in the context of natural selection imposed by the neuronal microenvironment, the imbalance will become amplified, leading to loss of both Type I neurons, due to their selective advantage, and Type II neurons, due to increased oxidative stress (Figure 14).

(iii) *Inverse Comorbidity*

Conditions for the inverse comorbidity of cancer and AD can be described in terms of the following properties:

- Epidemiological: The age incidence rate of the two diseases have similar statistical distributions
- Biochemical: If the enzymes in the metabolic pathways in two populations of individuals of the same age are subject to regulatory changes in the same direction, and generate negative correlated metabolic effects, then the probability of individuals being diagnosed with the diseases will be inversely related.

Although the amyloid cascade model may be consistent with the biochemical criterion, it is evidently inconsistent with the epidemiological condition. It is an established fact that the sporadic forms of most cancers are described by an age-incidence rate which increases exponentially with age. However, according to the amyloid cascade model, the age-incidence of AD will be a bell-shaped distribution. This implication is inconsistent with the epidemiological fact that cancer and AD are inversely comorbid.

The Inverse Warburg hypothesis, however, is consistent with both biochemical and epidemiological criteria. The consistency with biochemical conditions derives from the metabolic characterization of the Warburg and the Inverse Warburg effects. The Warburg effect can be considered an oncologic phenomenon. It pertains to the up-regulation of glycolysis to compensate for the impairment in energy production due to mitochondrial dysfunction in differentiated cells.

The Inverse Warburg effect can be considered a neuroenergetic phenomenon. It refers to the *up-regulation* of oxidative phosphorylation to compensate for the impairment in energy production due to mitochondrial dysfunction in neuronal cells. The glycolytic enzyme PFKFB3 is a critical directive in both modes of metabolic reprogramming. In cancer cells, the metabolic reprogramming involves an up-regulation of the enzyme PFKFB3 (Yalcin et al., 2009). In neurons, as the activity of the glycolysis-promoting enzyme PFKFB3 is maintained low by its constant proteasomal degradation, activation of glycolysis is prevented, thus favoring an enhancement of oxidative phosphorytion to face increased energy needs (Bolanos et al., 2010).

An increased glycolysis will involve an up-regulation of this key glycolytic enzyme and results in a high risk for the unlimited proliferation that defines the cancer phenotype. An increased OxPhos activity in the context of a lack of activity of the same key glycolytic enzyme would result in a high risk for the neuronal loss which characterizes the Alzheimer phenotype.

A strong activity of such a key enzyme will increase the risk of cancer but decrease the risk of AD. We can infer from these observations that cancer and AD will be inversely comorbid. This prediction is consistent with the epidemiological studies contrasting the incidence of both diseases. The consistency of the Inverse Warburg hypothesis with the epidemiological criteria is a result of the fact that age generates pathogenesis as a result of the *cumulative* increase in molecular disorder and metabolic dysregulation. Table 2 describes and contrasts the two mechanisms for AD, the amyloid hypothesis and the Inverse Warburg model, in the light of the demographic and epidemiological properties we have delineated.

**Table 2:** **Amyloid cascade hypothesis and Inverse Warburg hypothesis**.

**Properties**	**Amyloid cascade hypothesis**	**Inverse Warburg hypothesis**
Neuronal vulnerability	Random and non-selective	Selective
Age-specific disease incidence rate	Bell-shaped	Exponential increase with age
Comorbidity	Absence of comorbidity	Inverse comorbidity

## The energetic selection model: empirical considerations

There exists a body of empirical studies which investigate the incidence of the up-regulation of OxPhos activity as a compensatory response to impairment in energy metabolism as a result of genomic or metabolic factors. These investigations have been carried out in both cell culture contexts and in transgenic mouse models. We will appeal to these studies in furnishing empirical support for the Inverse Warburg effect.

### The up-regulation of OxPhos activity: cell culture studies

Empirical support for this mode of metabolic reprogramming is based largely on studies of hippocampal neurons—the cell type which is most vulnerable to mtDNA damage and impairment in metabolic dysregulation (Nagy et al., 1999; Zhu et al., 2006).

Zhu et al. (2006) have observed that levels of mtDNA and cytochrome oxidase (COX1) are markedly increased in hippocampal neurons of Alzheimer's patients even though the number of mitochondria per neuron is decreased. The increase in COX activity can be interpreted as the up-regulation of OxPhos activity—a compensatory action to mitigate the effect of diminished energy production in the highly damaged mitochondria. Nagy et al. (1999) have also observed an increase in COX1 activity in the mitochondria of neurons which have a diminished energy production. In these studies, neuronal COX activity increases with increasing age and shows a decline only with very old age. The increase in COX activity is consistent with the up-regulation of OxPhos as a compensatory mechanism. The decrease at very old ages reflects the loss of neuronal connections at advanced stages of the disease, that is, with the accumulation of AD-related pathology. Comparable decreases at very old age has also been documented by Chandrasekeran et al. (1998).

These experimental observations indicate that the up-regulation of OxPhos activity in neurons is a common mechanism to compensate for diminished energy production induced by mitochondrial dysfunction.

### Up-regulation of OxPhos activity: transgenic mice

Reddy et al. (2004) investigated the up-regulation of mitochondrial genes as a compensatory response to impaired energy metabolism by using an APP transgenic mouse model (the Tg2576 mouse model). The experimental studies focused on gene expression profiles at three stages of the disease progression. The stages considered were 2 month old mice (long before amyloid pathology), 5 month old mice—immediately before amyloid pathology, and 18 month old mice (after amyloid pathology and cognitive impairment). The analysis showed that the genes which are related to mitochondrial energy metabolism were up-regulated already in 2-month old mice, preceding the cognitive impairments that will appear later with age. The claim that this up-regulation is a compensatory response to mitochondrial impairment was based on studies of changes in cytochrome oxidase which was significantly decreased. Additional support for the hypothesis was based on changes in Complex (I), (III), and (IV). Complex (III) and (IV) both showed an increase in mRNA expression in both early and definite AD patients, a change associated with a great demand on energy production.

## Therapeutic implications: suppressing the inverse warburg effect

The metabolic stability theory of aging contends that the increase in probability of death associated with the aging process is the result of the cumulative effects of random perturbations on mitochondrial function (Brink et al., 2008).

These perturbations will ultimately induce metabolic dysregulation in some neurons and result in compensatory up-regulation of OxPhos—the Inverse-Warburg effect—ultimately contributing to the pathogenesis of AD. Accordingly, AD is an age-related disease and the transition toward the various hallmarks of the disease is a consequence of the aging process.

The model predicts that the transition toward AD is determined by the intensity of natural selection. Accordingly, impeding this transition may be modulated by regulating this evolutionary force. There are two principal strategies for reducing selection intensity:

(1) Enhancing the available concentration of oxidizable substrates such as lactate: neurons most likely utilize lactate as an important energy source under conditions of cellular stress. Lactate is produced by glycolysis in the astrocytes. An increase of glycolytic activity in astrocytes will increase the abundance of the energy source lactate, and thereby reduce the selective advantage of Type II cells. Alternatively, ketone bodies can also represent alternative oxidizable substrates for neurons. Enchancing the availability of these substrates could be achieved by different approaches. For example:
  (a) Pharmacological agents enhancing the activation of ionotropic glutamate receptors expressed by astrocytes (e.g., ampakines) have been shown to enhance the glycolytic response of astrocytes to glutamatergic activity (Pellerin and Magistretti, 2005).
  (b) The glycolytic phenotype of astrocytes can be enhanced by pharmacological agents inhibiting proline hydroxylases (e.g., Dimethyloxalylglycine), leading to a stabilization of the transcription factor HIF-1α in astrocytes that activates expression of several key enzymes and transporters involved in glycolysis (Brix et al., 2012; Rosafio and Pellerin, 2014).
  (c) Ketogenic diets. Ketone bodies have been shown to be adequate alternative oxidative energy substrates for the brain and to provide beneficial effects in certain pathological conditions that include epilepsy but also neurodegenerative diseases such as Alzheimer's disease (Gasior et al., 2006) as well as in preventing cognitive decline with aging (Balietti et al., 2010). Moreover, astrocytes have been shown to have the capacity to produce ketone bodies (Guzman and Blazquez, 2001). Just as endogenously produced lactate, they may alleviate the selective advantage of Type II neurons.

(2) Modulating the damage due to the collapse of homeostasis in ROS: both very high and very low concentration of ROS can have deleterious effects and thereby drive the system toward a state in which neuronal dysfunction is propagated to nearby neurons.
  (a) The effect of high concentration of ROS: This condition can be diminished by the upregulation of antioxidant enzymes, e.g., catalase or superoxide dismutase.
  (b) The effect of low concentration of ROS: Cell signaling is compromised when concentration of ROS is significantly reduced. Thus upregulation of antioxidant activities must be done cautiously to avoid disruption of oxidant signaling, with a focus on strategies to maintain stable levels of ROS rather than to achieve maximal reduction of ROS levels.

The theory we have developed in this article provides an evolutionary rationale for the possible effectiveness of these therapeutic strategies, and suggest furthermore how related target enzymes and/or metabolic pathways may have similar therapeutic effects. Thus, our hypothesis of the role of the Inverse Warburg effect in AD leads to testable predictions regarding novel therapeutic strategies for AD.

The inverse Warburg effect postulates that the up-regulation of OxPhos activity initiates the neurodegenerative cascade. This up-regulation of OxPhos has a metabolic origin—mitochondrial dysregulation induced by the process of aging. The therapeutic strategies which the metabolic perspective advocate is neuroglial. These strategies involve methods to enhance glial lactate supply thereby decreasing the selective advantage of unhealthy neurons. These features and the diagnostic programs which the two classes of models describe are summarized in Table 3.

**Table 3:** **Amyloid cascade hypothesis and the neuroenergetic model**.

**Properties**	**Amyloid cascade model**	**Inverse Warburg effect**
Primary cause	Over production of Beta amyloid	Upregulation of OxPhos
Diagnostic strategy	Test for beta amyloid	Test for increased OxPhos
Therapeutic strategy	Drugs to reduce Toxicity of beta Amyloid	Drugs to Enhance glial lactate supply Accentuate glial glycolytic phenotype Control antioxidant therapy

In order to be able to test some of the predictions made based on the Inverse Warburg hypothesis, it would be quite useful to have access to an appropriate animal model. Since an enhanced OxPhos activity would be not only a hallmark but also a causative factor of the process leading to neurodegeneration in the context of Alzheimer's disease, development of a mouse model exhibiting such a feature would be useful. The peroxisome proliferator-activated receptor γ coactivator PGC-1α is a key component involved in the regulation of mitochondrial biogenesis. Enhancing mitochondrial biogenesis via an overexpression of PGC-1α might be a good strategy to obtain an enhanced OxPhos activity in the central nervous system and particularly in neurons. Of note, a transgenic mouse overexpressing PGC-1α in all tissues has already been generated (Liang et al., 2009). Interestingly, when such mice were crossed with the Tg 19959 transgenic mouse model for Alzheimer's disease, it worsened the phenotype by causing mitochondrial abnormalities, exarcerbating amyloid and tau accumulation, inducing neuronal death as well as provoking behavioral abnormalities (as expected according to the Inverse Warburg hypothesis) instead of improving it by enhancing OxPhos activity (as postulated based on the hypothesis that β-amyloid reduces OxPhos activity) (Dumont et al., 2014). Such trangenic mice aiming at selectively enhancing OxPhos activity in neurons could represent excellent models to test experimentally the validity and usefulness of the Inverse Warburg hypothesis, especially if examined in the context of aging.

## Conclusion

Alzheimer's disease is a neurodegenerative disorder, characterized by neuronal loss and progressive cognitive decline leading to dementia. This article explains how the up-regulation of OxPhos acivity in impaired neurons and competition for a limited resource (astrocyte-derived lactate as a substrate for neuronal OxPhos) can lead to deleterious effects on initially healthy neurons in the vicinity of other neurons that have mitochondrial dysfunction. The model predicts that the severity of the disease will be a consequence of the intensity of natural selection involving two classes of neurons: Type I neurons, cells with normal levels of OxPhos activity, and Type II neurons, cells with mitochondrial dysfunction and compensatory increases in levels of OxPhos.

Our analysis predicts that the intensity of selection, which depends largely on the capacity of astrocytes to supply sufficient amounts of energy substrates, can be reduced by metabolic interventions that: (a) alter the composition of the neuronal microenvironment by increasing astrocytic lactate production, such that lactate is no longer a limiting resource; and (b) reduce the deleterious effects of large variations in ROS by the cautious use of antioxidants to modulate the effects of high ROS.

This article has contrasted the amyloid cascade model—a genetic, neuroncentric depiction of the origin and development of AD, with a neuroenergetic model—a metabolic, neuron-glial depiction of AD. The contrast is analyzed in terms of certain classes of empirical observations which have been observed in studies of AD. These empirical observations are consistent with the neuroenergetic model but inconsistent with the amyloid hypothesis.

### Conflict of interest statement

The authors declare that the research was conducted in the absence of any commercial or financial relationships that could be construed as a potential conflict of interest.
